# Immune Cells and Transcriptional Signatures Revealed Novel Regulators and Predict Clinical Response to Biologic Therapy in Ulcerative Colitis

**DOI:** 10.33696/immunology.3.116

**Published:** 2021

**Authors:** Suzana D. Savkovic

**Affiliations:** Department of Pathology and Laboratory Medicine, Tulane University; 1430 Tulane Ave SL-79; New Orleans, LA 70112 USA

Inflammatory Bowel Disease (IBD), which includes Crohn’s Disease (CD) and Ulcerative Colitis (UC), has a heterogeneous pathogenesis underlined by genetic predisposition, intestinal barrier dysfunction, impaired immune response, and microbiota imbalance [1-3]. This proceeds to aberrant immune cells presence and function in the affected tissue, activation of signaling pathways, and expression of regulators that subsequently drive inflammation [2,4-7]. Using publicly available transcriptomes obtained from large number of UC patients from European and the US cohorts [8-16], we identified systemic immune cell landscape, pathways, and transcriptional signatures specific for UC as well as those determining outcome of biologic therapy [17].

The global immune cell landscape in UC colonic tissue, determined by CIBERSORT [18] assessment of transcriptomes, revealed systemically elevated levels of neutrophils, T CD4 memory activated cells, active dendritic cells (DC), M0/M1 macrophages, and B naïve cells. Several cell subsets were noticeably reduced such as T CD8, Tregs, B memory, and M2 macrophages. In addition, both resting DC and resting mast cells were lowered, while their active forms were elevated in UC tissue [17]. Relative abundances of other cell subsets in UC tissue were also altered but did not meet significant threshold with applied criteria. Individually, these immune cells are recognized to have important roles in intestinal inflammation [4-6]. Our findings demonstrated that this systemic immune cell landscape was common across UC patients in multiple cohorts. Elevated level of neutrophils in intestinal tissue, an early sign of inflammation, fuels disease by augmenting tissue damage [6,19]. Aberrant T cells and macrophages in the intestine are critical for facilitating inflammatory responses and injury [4,5,20,21]. Some immune cells have dual functions, such as B cells and DC, which are initially protective, but in the long run contribute to UC pathobiology [22-24]. Further, immune cells including macrophages can foster each other’s activity and stimulate other immune cells in promoting inflammation [5,25-27]. Moreover, decrease in certain cells in the intestine impairs protection from bacterial products from the lumen and weakens antigen presentation and processing [28]. Furthermore, in uninflamed (matched) colonic tissue from patients with active UC the majority of the samples displayed an immune cell landscape similar to healthy colonic tissue. However, presence of certain cells (CD4 memory activated and Tregs) was similar to inflamed UC tissue, while presence of other cells (subsets of DC and macrophages) differed from both healthy and inflamed UC. Thus, uninflamed colonic tissue from patients with active UC may provide important information about pathogenesis and recurrent inflammatory episodes. Additionally, we found that a subset of T CD4 cells differed between cohorts, suggesting that these cells may be responsible for disease relapse [21,29]. We speculate that this difference between patient cohorts could be due to variations in composition of microbiota and diversity of regional diet. Similarly, differences in eosinophils, which are involved in protecting intestinal barrier integrity and immunity, might be related to geographic and seasonal disparities among UC cohorts [30,31]. Furthermore, UC tissue obtained from patients prior to biologic therapy with anti-TNFα and anti-α4β7, which were later identified clinically as non-responders, had considerably more neutrophils and T CD4 activated cells when compared to responders. Clinical studies demonstrated that non-responsiveness to the biologic therapy is, in part, related to disease severity, patients age at diagnosis, and duration of inflammation [32-35]. Similar findings are recently reported using single-cell sequencing of a UC cohort [36]. The single-cell sequencing approach provides insight into the existence and behavior of different cell types, while CIBERSORT allows analysis of transcriptomes from multiple cohorts as well as from already existing transcriptomic data. This supports the importance of different approaches to understand the complexity of cell composition in UC pathobiology.

Using Ingenuity Pathway Analysis, we discovered signaling pathways associated with differentially expressed genes (DEGs) in UC tissue compared to controls across different cohorts [17]. These systemic pathways are linked to bacterial response, inflammation, and intracellular signaling. Further, we identified a transcriptional signature consisting of the top hundred DEGs (UC_100_) that are common across three different cohorts [17]. In an independent cohort, the UC_100_ distinctly separated inflamed from non-inflamed samples via hierarchical clustering. Among the DEGs in the UC_100_ are those with established roles in UC pathobiology including regulators of hypoxia, nitric oxide, inflammation, matrix metallopeptidases, and calcium signaling [6,37-40]. Moreover, we selected DEGs whose role in IBD are not well examined for validation in primary UC tissue (by qPCR) that encode regulators of lipid metabolism and mitochondrial function. As for regulators of lipid metabolism, we found increased LPCAT1, which controls lipid droplet number and size [41], LIPG, involved in lipoprotein metabolism [42], and HCAR3, which regulates lipolysis [43,44]. Increased lipid metabolism and intracellular lipid droplets drive intestinal inflammation [45-47]. In addition, LIPG may be involved in endothelial biology [42] and HCAR3 may play roles in crosstalk between metabolites derived from microbiota and immune cells [43,44]. Moreover, DEGs encoding regulators of mitochondrial function including ACAT1 and HMGCS2 are decreased in UC tissues. Limited studies demonstrated that loss of HMGCS2 function in intestinal stem cells could impact intestinal barrier renewal and function [48,49], while ACAT1 has been recently implicated in inflammatory responses in macrophages [50]. In IBD, aberrant expression of mitochondrial regulators leads to reduced respiratory activity, which may further exacerbate response to bacterial signaling [51-55]. A recent study revealed that mitochondrial fission-fusion is critical in homeostasis of intestinal cells and macrophages [56]. Mitochondrial reprograming may also depend on environmental factors, such as use of antibiotics and intake of a high-fat western diet [57]. The exact mechanisms and role of metabolic reprograming with lipids and mitochondria in intestinal cells and immune cells are not fully understood. Their emergence as a hallmark of intestinal inflammation highlights the importance of lipids and mitochondria in the underlying mechanism of disease.

Furthermore, twenty “resistant” DEGs (UC_20R_) from transcriptomes of UC patients that were common for non-responders to both anti-TNFα and anti-α4β7 therapy were identified [17]. DEGs within UC_20R_ encode regulators involved in bacteria response, defense response, cell signaling, cell trafficking, endothelial function, and metabolism. The UC_20R_ transcriptional signature had significant predictive power for determining (non) response to both anti-TNFα and anti-α4β7 therapy as demonstrated by receiver operating characteristic (ROC) curve analysis and calculating area under the curve (AUC) [17]. Several DEGs with the highest prediction (sensitivity of 73.3% and specificity 85.7%) for non-responding to both therapies, including SELE, VNN2, and STC1, have critical roles in neutrophil accumulation and transendothelial movement at sites of inflammation [58]. This suggests a possible role of immune cell trafficking in predicting response to biologic therapy. Moreover, these features of disease could also be, in part, mediated by changes in the microbiota caused by therapy [59]. Therefore, identifying additional resistant signatures in UC patients may provide guidance for using select therapy and more personalized therapeutic approaches.

A comprehensive assessment of transcriptomes from UC patient colonic tissue demonstrated shared and distinct immune cell landscape, signaling pathways, and transcriptional signatures among cohorts. Further development of new approaches differentiating active from non-active immune cells and interactive from non-interactive cells may provide a platform for more precision-based identifiers of cell heterogenicity in affected tissue. Additionally, combination of bioinformatics approach with human genetics, epigenetics, and single-cell genomics will lead to mechanistic understanding of inflammatory disorders, risks of recurrence, and association with treatment outcomes. Consequently, it is plausible that these directions may provide clues for development of more precise, personalized diagnostics and therapeutic intervention for UC.
